# Continuous Non-Invasive Glucose Monitoring via Contact Lenses: Current Approaches and Future Perspectives

**DOI:** 10.3390/bios11060189

**Published:** 2021-06-09

**Authors:** David Bamgboje, Iasonas Christoulakis, Ioannis Smanis, Gaurav Chavan, Rinkal Shah, Masoud Malekzadeh, Ioannis Violaris, Nikolaos Giannakeas, Markos Tsipouras, Konstantinos Kalafatakis, Alexandros Tzallas

**Affiliations:** 1 Department of Electrical & Computer Engineering, University of Massachusetts Lowell, Lowell, MA 01854, USA; Olorunfemi_Bamgboje@student.uml.edu (D.B.); Ioannis_Smanis@student.uml.edu (I.S.); Gaurav_Chavan@student.uml.edu (G.C.); rinkal_shah@student.uml.edu (R.S.); Masoud_Malekzadeh@student.uml.edu (M.M.); 2 Department of Informatics and Telecommunications, School of Informatics and Telecommunications, University of Ioannina, 471 00 Arta, Greece; iasonasch@gmail.com (I.C.); giannakeas@uoi.gr (N.G.); k.kalafatakis@uoi.gr (K.K.); 3 Department of Electrical and Computer Engineering, University of Western Macedonia, 501 31 Kozani, Greece; john.violaris@yahoo.com (I.V.); mtsipouras@uowm.gr (M.T.)

**Keywords:** contact lenses, glucose sensor, biosensors, low power, wireless health monitoring, non-invasive monitoring

## Abstract

Diabetes mellitus (DM) is a chronic disease that must be carefully managed to prevent serious complications such as cardiovascular disease, retinopathy, nephropathy and neuropathy. Self-monitoring of blood glucose is a crucial tool for managing diabetes and, at present, all relevant procedures are invasive while they only provide periodic measurements. The pain and measurement intermittency associated with invasive techniques resulted in the exploration of painless, continuous, and non-invasive techniques of glucose measurement that would facilitate intensive management. The focus of this review paper is the existing solutions for continuous non-invasive glucose monitoring via contact lenses (CLs) and to carry out a detailed, qualitative, and comparative analysis to inform prospective researchers on viable pathways. Direct glucose monitoring via CLs is contingent on the detection of biomarkers present in the lacrimal fluid. In this review, emphasis is given on two types of sensors: a graphene-AgNW hybrid sensor and an amperometric sensor. Both sensors can detect the presence of glucose in the lacrimal fluid by using the enzyme, glucose oxidase. Additionally, this review covers fabrication procedures for CL biosensors. Ever since Google published the first glucose monitoring embedded system on a CL, CL biosensors have been considered state-of-the-art in the medical device research and development industry. The CL not only has to have a sensory system, it must also have an embedded integrated circuit (IC) for readout and wireless communication. Moreover, to retain mobility and ease of use of the CLs used for continuous glucose monitoring, the power supply to the solid-state IC on such CLs must be wireless. Currently, there are four methods of powering CLs: utilizing solar energy, via a biofuel cell, or by inductive or radiofrequency (RF) power. Although, there are many limitations associated with each method, the limitations common to all, are safety restrictions and CL size limitations. Bearing this in mind, RF power has received most of the attention in reported literature, whereas solar power has received the least attention in the literature. CLs seem a very promising target for cutting edge biotechnological applications of diagnostic, prognostic and therapeutic relevance.

## 1. Introduction

Diabetes mellitus (DM) is a metabolic disease characterized by raised blood glucose levels, which in the long-term cause complications, leading to microvascular disease, increasing the risk of stroke or ischemic heart disease or peripheral vascular disease (due to development of atherosclerotic plaques), retinopathy (potentially leading to blindness), nephropathy (leading to chronic renal failure) and/or neuropathy. The World Health Organization (WHO) indicates that more than 220 million people live with DM globally. DM has been the underlying cause of about 1.5 million deaths in 2012, and this number is estimated to worsen by 100% until 2030 [1,2]. Early diagnosis and tight control of blood glucose concentrations are crucial for these patients to avoid short- and long-term complications [3].

Close monitoring of glucose is of paramount importance in diabetic patients, since their body cannot automatically and quickly compensate for the increases and decreases of the systematic glucose levels resulting from the frequent presence of relevant cues with opposing effects during each day. For instance, eating sessions, oscillations of hormones affecting glucose mobilization and metabolism, oscillations in the metabolic capacity of organs, stress events, and the administration of drugs directly or indirectly affecting glucose availability and consumption. Nevertheless, glucose must remain within a relatively narrow range of values; lower levels (hypoglycemia) might acutely endanger neuronal cell viability, a life-threatening condition, while high levels of glucose might also cause potentially serious health problems. These include diabetic ketoacidosis or hyperosmolar hyperglycemic state in the short term, and permanent vascular and neurotoxic damage in the long run. Continuous glucose monitoring combined with rapid corrections of any glucose level deviations might significantly improve DM therapeutics in minimizing the hypo- and hyperglycemic episodes that compromise homeostasis.

The most commonly used technology for blood glucose determination is an enzyme-based method, which requires frequent blood sampling and therefore blood drawing. While “finger pricking” is a relatively painless process, this method does suffer from a few practical problems. The first one is the inconvenience and required compliance by patients, while the second is that this is not a continuous monitoring method [4]. Furthermore, the reports also indicate that the use of an even minimally invasive biosensor cannot completely exclude the possibility of a blood-borne infection. Raman spectroscopy, infrared spectroscopy, fluorescence spectroscopy and surface plasma resonance constitute other techniques used for glucose measurement, but these techniques are also not useful for continuous glucose monitoring. Other body fluids, besides plasma, have therefore been utilized and have enormous potential for non-invasive continuous glucose monitoring; among them, saliva, urine and lacrimal fluid (tears). Compared to other body fluids, tears are more accessible than blood or interstitial fluid, more continuously obtainable than saliva, and less susceptible to dilution than urine [5,6,7].

Contact lenses (CLs) have a promising potential for being used as glucose sensors, while also playing an important part in lubricating the eyes and nourishing the cornea. In the standard conditions of humidity and temperature, the average rate of lacrimal fluid production is 2.2 µL/min. Sensors are embedded between two soft layers of lens material and a pinhole in the lens allows lacrimal fluid to seep into the sensor and be used to measure glucose levels. A wireless antenna, thinner than a strand of human hair, will act as a controller to communicate information to the wireless device. Data will then be sent to an external device. CLs can analyze glucose levels every second and transmit the data to an associated App. CLs could potentially diminish the problem of dynamic monitoring of glucose levels to determine and correct unwanted glucose peaks or troughs during the day and night, achieving a much tighter control of the hormonal oscillations.

Evidence on the rationale for utilizing lacrimal fluid glucose has been provided, in parallel with efforts on developing reliable, quantitative methods for its estimation. March (2001) performed concurrent, invasive measurements of plasma and anterior aqueous humor glucose concentrations on rabbits over a period of several weeks, showing the existence of a correlation of glucose levels between the blood stream and the eyes [8]. Later on, Domschk et al. (2006) determined the effectiveness of a new holographic CL-based glucose sensor for the non-invasive monitoring of blood glucose, in which the hologram was created as an acrylamide copolymer hydrogel with phenyl boronated groups acting as reversible binding ligands. When glucose is bound to these ligands, the interference fringes swelled, changing the color of the light reflected off the hologram. This color change is used to quantify glucose concentrations [9].

In this review paper, we aim at comprehensively summarizing the up-to-date key enabling technologies that allow for a reliable CL-based detection of lacrimal fluid glucose levels. Initially, we present an overview of the sensing methods and principles used (focusing on graphene-based and amperometric glucose sensors). Subsequently, we focus on system integration, and thereafter, on signal transmission, which includes radiofrequency (RF) and optical transmission, and on system performance evaluation. The next part of the review covers in detail the concept behind the various approaches of wireless power distribution to CLs (specifically, techniques such as RF power, biofuel cell, solar cell, and inductive power). Future medical applications of CLs, such as ultraviolet protection (UV) and the application of epithelial stem cells in curing blindness, are briefly also highlighted. Finally, a statistical estimate of CL power resource research coverage is presented, and some key conclusions are drawn.

## 2. Sensing Methods and Principles

### 2.1. Graphene Hybrid Glucose Sensor

**Figure 1:**
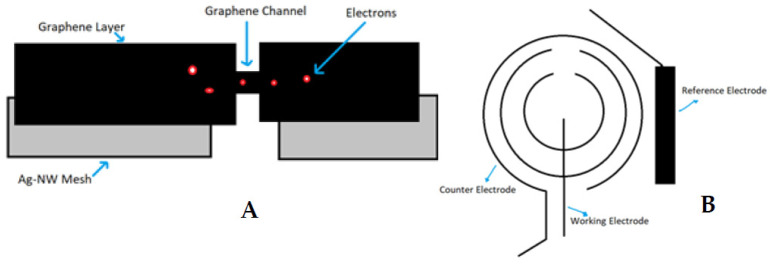
Glucose-sensing principles for contact lenses; graphene hybrid glucose sensor (**A**) and amperometric glucose sensor (**B**).

Field-Effect Transistor (FET)-based glucose sensors that consist of the graphene channel and hybrid source/drain (S/D), have the potential to detect glucose levels in a non-invasive manner. The graphene-AgNW hybrid film is formed by transferring graphene onto random networks of AgNW (Figure 1A). The hybrid has significantly reduced sheet resistance, with slightly lower optical transmittance and haziness compared to the single material of graphene or AgNW. All device components are transparent, with a slightly visible spiral antenna, where graphene channels are integrated onto the wearable CL. Glucose oxidase (GOD, b-D-glucose from Aspergillus Niger) is immobilized on the graphene channel using a pyrene linker via π-π stacking for selective and sensitive detection of glucose molecules. GOD is attached to the pyrene linker by the amide bond from the nucleophilic substitution of N-hydroxysuccinimide. To confirm the selective binding of GOD on the surface of the graphene channel, atomic force microscopy (AFM) is used. The detection mechanism of this sensor includes the catalysis of glucose oxidation by GOD (to gluconic acid) and the reduction of water to hydrogen peroxide. Hydrogen peroxide is oxidized to produce oxygen, protons, and electrons (H_2_O_2_ → 2H^+^ + O_2_ + 2e^−^). The concentration of the produced electrons generates an electrical current in the channel, and thus the drain current increases proportionally with the concentration of glucose [10].

More recently, a modified version of that graphene-AgNW film has been described [11]; the method utilizes the synergistic combination of one-dimensional Ag-NWs and two-dimensional graphene sheets in the form of highly porous 3D nanostructures. To achieve this, a simple hydrothermal sol-gel technique was applied by establishing covalent crosslinks.

Other graphene-based glucose sensors include (i) the single-wall carbon nanotubes/cuprous oxide/zinc oxide nanorods/graphene hybrid electrodes, and (ii) the three-dimensional (3D) graphene–copper hybrid electrodes. For the synthesis of the former, the graphene was grown by chemical vapor deposition, subsequently wet-transferred onto indium transparent oxide glass, on which the zinc oxide seed layer was sputtered, while zinc oxide nanorods were gradually grown by the hydrothermal method. Finally, the zinc oxide nanorods were clad with cuprous oxide by the electrochemical method [12]. On the other hand, the 3D graphene-copper film was synthesized onto a 3D porous nickel foam substrate by chemical vapor deposition and a thin copper layer was evaporated onto the 3D graphene/nickel foam by thermal evaporation [13].

### 2.2. Amperometric Glucose Sensor

Similarly, the amperometric glucose sensor detects electrons generated after reaction with glucose in the lacrimal fluid. The GOD is immobilized in a Titania sol-gel layer. The sensitivity of the sensor can be enhanced by altering the properties of the sol-gel to satisfy the low-level detection requirement for glucose determination in lacrimal fluid. Three electrodes (working electrode, counter electrode and reference electrode) are used to detect the presence and amount of glucose molecules in the lacrimal fluid and then determine the electric current by measuring the cumulative electron charge. The principle of sensing the presence of glucose is similar to the graphene hybrid glucose sensor, as glucose gets oxidized by GOD to yield hydrogen peroxide and D-gluconolactone. Then, the D-gluconolactone reacts with water to give gluconic acid, and the electrochemical oxidation of hydrogen peroxide generates two free electrons. These electrons are detected by the working electrode. To integrate the glucose sensor on a CL, the sensor must be designed according to normal CL dimensions and be fabricated on a 10 µm thick film of flexible and transparent polyethylene terephthalate (PET) polymer. The biosensor is composed of a working, a counter, and a reference electrode (Figure 1B). The working and counter electrodes are designed as concentric rings to minimize the electrical resistance between them. The bare plastic surface between the working and counter electrode is used as a platform to immobilize glucose oxidase (GOD). The reference electrode is designed as a rectangular bar (1.6 mm × 0.25 mm) near the sensing area [14].

Modifications of this approach use a thin film of poly-hydroxylmethylmethacrylate as the immobilization agent for GOD [15], or use two working and two conjoint counter electrodes with one common reference electrode [16,17], or different combinations of elements to fabricate the sensor (either a combination of titanium, palladium and platinum or a combination of platinum and iridium) [18].

### 2.3. Photonic Sensor—A Variation of Amperometric Sensors

Another variation of glucose sensors is the photonic sensor (PS), which is based on the reversible covalent interaction of hydrogel. In this case glucose-selective hydrogel film uses phenylboronic acid to bind glucose as mentioned above. But this time the hydrogel layer uses the reversible swelling/shrinking effect to change its thickness proportionally to the glucose concentration. Using optical near infrared spectroscopy, a correlation has been observed between the diffraction of hydrogel and concentration of glucose within 0–50 mM. Diffraction is a known optical technique to analyze the properties of the microstructure of a nanolayer. The reflected power of first-order diffraction was processed via a smartphone. So, glucose-sensitive PS may be useful in continuous hormonal monitoring of DM patients, excluding those who suffer from eye diseases that change the intraocular pressure. This technique offers: (i) no need of enzymes or circuits, (ii) the capability of analyzing the diffraction using a smartphone without the presence of a photodetector and (iii) the usage of contact lenses which are commercially available [19,20].

### 2.4. Optical Sensing Methods

Fluorescent sensor: Another optical sensor that can monitor the glucose concentration in tears is called a fluorescent sensor. It uses fluorescence probes to monitor the blood glucose levels via its transduction elements (boronic acid containing fluorophores) which respond in the presence of glucose. These elements induce a chemical phenomenon that leads to spectral changes (i.e., a fluorescent detection system), based on the conformation of boronic acid from neutral to anionic. A fluorometer is used to perform the measurements. Nevertheless, the reliability of glucose estimations is low beyond a relatively narrow spectrum; incompatible pH conditions exist between the CL microenvironment and the optimal functioning detection solution, leading to reduced detection sensitivity when the system is incorporated into the CLs, and there is a variable response time depending on the lifetime of the biosensor, which together, eliminate its usefulness [21,22].

Spectroscopy sensor: Another application of the glucose monitoring sensor recruits an optical approach. Kim et al. deployed nanoparticles embedded on a contact lens (NECL). This approach requires no external power to make measurements. It uses a spectrometer system working in reflectance mode. The emission of light comes through a sphere that conducts the reflection of a sample to the detector of the spectrometer. The interior coating of the sphere is made of polytetrafluoroethylene, which has up to 95% reflectivity in the visible region of the spectrum. The NECL showed off less than 6% of relative reflectivity. Disadvantages in this sensing method are: the complex output analysis pipeline, requiring pre- and postprocessing steps; the complex fabrication technique; the barely zero transmittance, and potential disruption of visual acuity [23].

Other optical sensing methods: Different phenomena of light can detect glucose qualitatively and quantitatively, by utilizing the properties of light. Some of them are reflection, refraction or diffraction. For instance, kromoscopy involves the transmission of a broad band of electromagnetic interference (EMI) through the sample of interest. This transmitted light is analyzed by a four-channel detector providing a high signal to noise ratio [24]. Photoacoustic spectroscopy uses the light in operation with ultrasound waves. Irradiated tissues conduct ultrasound waves due to heat. The generated pulse is easily detectable. As the glucose concentration increases, the specific heat capacity of the tissue is reduced, and the RF velocity is increased [25]. Optical coherence tomography uses a low power laser; the synchronous light irradiates the sample, and the backscatter radiation is measured. Polarimetry on the other hand is based on rotation of the polarized light. This method is highly effective due to its independence from factors such as pH, temperature and wavelength [26]. Thermal infrared is a surrogate technique based on the dependence of transdermal microcirculation on the glucose concentration. Most of these techniques have hardly been incorporated into a CL-based biosensor, even for experimental purposes.

## 3. System Integration

### 3.1. Hardware Material Structure

Silicon (Si) diodes, capacitors, surface mount circuits and antennas, are practically impossible to integrate onto a severely constrained area such as a CL. Wireless data transmission from a device that cannot incorporate any type of battery comprises another challenge for CL sensor development. Current research is focusing on pioneering new material composites and polymer substrates, which could overcome all these technical challenges.

A particularly challenging domain for materials scientists who work on smart CLs is the development of biocompatible materials that have electrical properties such as adjustable capacitance, resistance, induction, while concurrently being able to deal with mechanical strain [27]. Material scientists chose to use hybrid substrates enhanced with conductive polymers such as polyethylene terephthalate (PET) bonding them with other materials such as Platinum (Pt), Silver Chloride (Ag/AgCl) and, graphene [27,28,29].

### 3.2. Eye Electromagnetic (EM) Waves Protection

Graphene is the most popular material used in the development of smart CL sensors, featuring a variety of benefits. Graphene’s two-dimensional lattice structure of carbon atoms is a key feature that may be used to protect human tissue and vital organs from being exposed to EM waves. Graphene’s grid can absorb the EM waves and convert them into thermal energy (heat) on its surface. It secures the interior human eye from serious damage by dissipating the heat. Graphene’s suitability for any smart CL was proved by its performance as an EM shield. Experiments which took place at Seoul National University provided convincing evidence about graphene’s high shielding properties. This component can therefore be the basis for various applications due to its ability to block high energy radiation [30].

### 3.3. Smart CL Sensor Types

**Figure 2:**
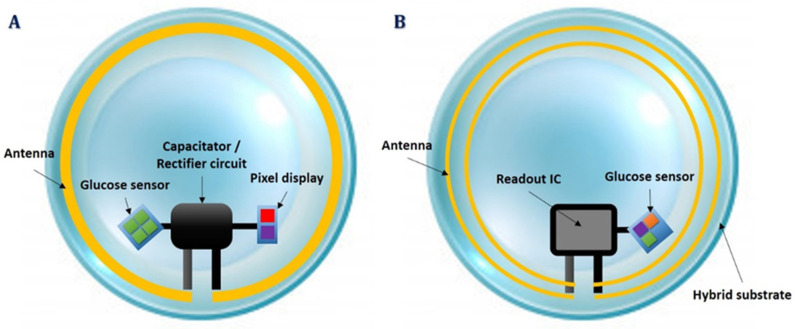
Smart contact lens integration systems; passive contact lens sensor circuit (**A**) and active contact lens sensor hardware (**B**).

There are two widespread types of smart CL sensors: the passive and the active ones. Passive CL sensors consist of few components, such as biocompatible capacitors, resistors, rectifiers, antennas, and microscale light-emitting silicon diodes (μLED). They are meant to function as simple-to-use visual indicators, having two binary states (on-off), depending on the resistance reductions. The higher the glucose levels, the lower the resistance, the higher the current which keeps μLED in the on state. On the contrary, at low glucose levels, thus high resistance, the current drops, and below a certain threshold the μLED is turned off. The μLED emits visible light, while a transparent and stretchable inductive antenna receives power from an external power transmitter. A rectifier circuit is used in this system to power up the rest of the circuit (Figure 2A). Advantages of this passive type of CL sensor include its cheaper cost and its fabrication simplicity. On the other hand, a passive CL sensor only returns visual information suggesting that a predefined glucose level threshold has been exceeded [31], and additionally, it only works for distances up to 5 mm from a power transmitter.

Active CL sensors potentially provide more essential benefits for real, continuous glucose monitoring. They also use the amperometric technique to measure glucose concentrations, incorporating built-in wireless electronics for continuous data transmission [27,28,29]. Moreover, active smart CL sensors involve a more complex circuit design and require more graphene layers to prevent heat transfer to the eye core. A tiny integrated circuit (IC), which is called a ReadOut IC, is required to pack power rectifiers, signal modulators, pulse generators, potentiostats and voltage regulators to operate at a constant voltage and frequency, until data transmission occurs [28,32]. The potentiostat is the electronic circuit required to control three electrode cells and run most electroanalytical experiments (Figure 2B). An active CL sensor can continuously estimate lacrimal fluid glucose concentrations, it can operate within a 15 cm distance from the power transmitter consuming only 3 μW, and is compatible with near field communication-enabled devices, such as smartphones. In turn, an active CL sensor has a more complicated fabrication process and involves integrated circuits of a bigger size.

## 4. Measurement Transmission and System Performance

Data transmission takes place only in the case of an active CL sensor, during the feedback calculation period. We define, as feedback calculation, the time, when the glucose-dependent resistance is measured from the sensing circuit area until the value is encoded and modulated to a low frequency RF signal and transmitted back to the user’s receiver device. Nevertheless, aside from RF transmission, there is another, optical technique to transmit data, described below.

### 4.1. RF Transmission

As a result of the extremely limited area that is available for circuit development on a CL, the lithography of the ReadOut IC does not go beyond 0.13 μm, packing all the essential components in a complementary metal–oxide–semiconductor (CMOS) package with an area of 0.36 mm^2^ [32]. This is the reason there is only one antenna that receives power in high frequency electromagnetic waves (between 1.5 Ghz and 2.5 Ghz) and then it is utilized to propagate lower frequency waves returning glucose measurements as modulated signals at 850 Hz.

### 4.2. Optical Transmission

**Figure 3:**
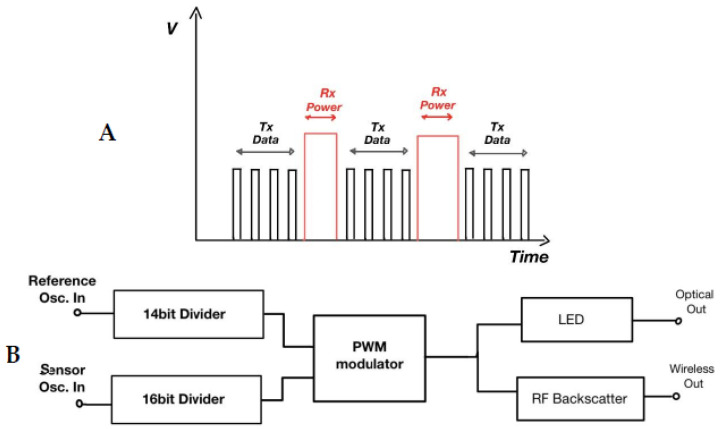
(**A**) μLED PWM data output pulses and induction power reception. (**B**) Measurements transmission via μLED and/or RF waves. LED: light-emitting silicon diodes, PWM: pulse-width modulation, Osc.: oscillation, RF: radiofrequency, Rx: receive, Tx: transmit.

Liao et al. [32] presented a great approach in smart CL sensor implementation that utilizes the μLED optical technique. They developed and implemented a CL application which is based on a complex circuitry that converts the frequency difference between two oscillating signals to measure the concentration of glucose in the tear fluid. The ReadOut IC generates two low-frequency signals, namely the Reference Oscillation and the Sensor Oscillation. The latter passes through the actual glucose sensor circuit and, depending on the glucose concentration, affects the output signal frequency (Figure 3B). The result of this frequency difference is modulated and propagated wirelessly to a specific type of RF receiver, or optically to a photosensitive sensor built in the receiver side which is usually a polymer substrate with incorporated RF antennas, induction coils and sensors. The proposed method for optical data transmission involves a μLED at the end of the signal modulator, that emits a sequence of short-period-width pulses after every induction pulse power reception. This is a well-known pulse width modulation signal (Figure 3A) with narrow duty cycles that drives a tiny-sized low-power LED bulb. The signal modulator generates a few (~3) μsec width pulses lowering the LED power to emit an optical signal in the visible spectrum. These pulses are generated in the first half period of the Reference Oscillation conserving even more energy and then the LED is set to off.

### 4.3. System Performance

The system integration combines both RF wireless and optical glucose measurements transmission in one system. Validation tests have been conducted on such a system to estimate its performance (i.e., test the sensitivity of the sensor in relation to glucose concentrations and the output signal frequency), utilizing low-power CLs, receiving 20 measurements with two different glucose systems, and implementing Alan deviation analysis. Results showed a strong linear correlation (r = 0.98) between glucose levels and signal frequency at 400 Hz/mM [32].

## 5. Power Delivery

To make a wearable glucose-detecting CL possible, the power supply to such a CL has to be wireless. Power is needed mainly for the IC components (Integrated Circuit) which will be on the CL [32]. Depending on the mode of glucose sensing, the CL could also contain LEDs which would also be powered [31,32]. Owing to the small size of CLs, about 1 cm^2^ with a thickness of about 0.2 mm [33], and safety restrictions [34], there is a limit to the size and number of components that can be incorporated onto a CL for power generation. This eliminates some conventional power sources such as batteries; however, credible power must still be supplied to the IC on the CL. To achieve credible power transfer to the CL, about four different approaches have been tried and are reported in the literature. Namely, RF power [31,32,35,36], biofuel cell [37,38,39], solar power [40] and inductive power [33,41,42].

### 5.1. RF Power

**Figure 4:**
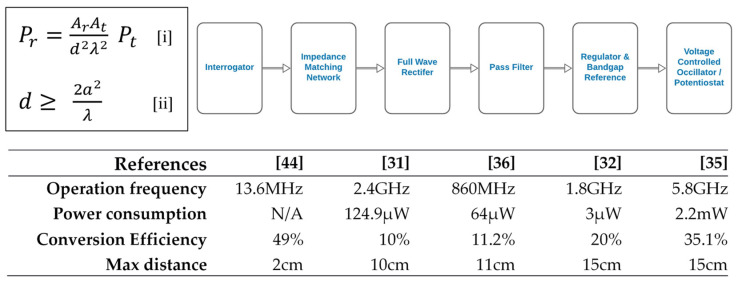
(**Upper left** panel) By characterizing an antenna by its effective area instead of its power gain or radiation resistance, an estimation of the maximum power that can be obtained at the receiving antenna of a radio circuit in free space, assuming a plane wavefront, can be obtained (equations [i] and [ii]). *Ar* is the effective area of the receiving antenna, *At* is the effective area of the transmitting antenna, *Pr* is the power at the receiving antenna, *Pt* is the power at the transmitting antenna, *d* is the distance between the transmitter and receiver, *λ* is the wavelength of transmitted signal, and *a* is the maximum linear dimension of either antenna, respectively. (**Upper right** panel) Stages of radiofrequency power transfer to contact lenses. (**Lower** panel) Performance comparison for radiofrequency-powered contact lenses [31,32,35,36,44]–it is evident that the best result was achieved by the Cheng group [35]. This is because they met the minimum safe distance of 11 cm [34] and had a high conversion efficiency.

This is the most popular way of powering a CL. The foundation of RF power stems from the classical work on a simple transmission formula [43] (Figure 4, upper left panel). Building on this foundation, the general layout of the stages in RF power transfer to CLs have been established in the literature (Figure 4, upper right panel). Basically, the interrogator sends high-frequency pulse energy through the impedance matching network which enables maximum power transfer to the rectifier for subsequent rectification. The DC output is then filtered and regulated to provide a stable reference for the voltage-controlled oscillator which sends sensor readings to the external reader [35]. The rectified output could also be used to power the potentiostat and/or pulse generator for subsequent sensor current measurement [31,32,36]. As interesting and popular as this approach of powering CL may be, there are numerous challenges to it. Some of them include EMI and switching noise, size restriction, fluctuating supply, complete transmitter and receiver integration, low efficiency, and process voltage temperature (PVT) effect. To solve some of these challenges, numerous methods have been proposed in the literature. To minimize EMI and reduce noise, a cleansing capacitor was used in many research studies [31,32,35,36]. The idea was to sort out the high-frequency signals. To reduce supply fluctuation, an ultra-low-power linear regulator, bandgap reference and bias current generation was used [32,34,36]. The idea was to provide the necessary isolation through a biased amplifier and a couple of low threshold voltage PMOS. In the work by Cheng et al., the problem of impedance mismatch and PVT effect was tackled by using a low loss and high Q factor LC network to yield a high resonant frequency and a high bandwidth, thereby minimizing impedance mismatch, PVT effect and hence, increasing the power conversion efficiency [35]. In a nutshell, the key features and power performance metrics of RF power application to CLs in the literature are shown in the lower panel of Figure 4 [44].

### 5.2. Biofuel Cell

**Figure 5:**
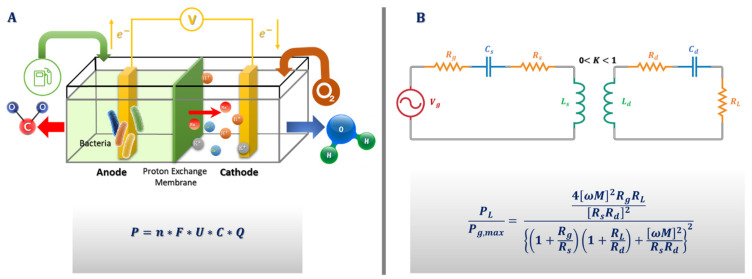
(**A**) Microorganisms producing energy as biofuel cells. P is the electric power, *n* the number of electrons released from one fuel molecule, *F* the Faraday constant, U the biofuel cell voltage, *C* the fuel concentration, and *Q* is the tear production rate. *U* values are based on the maximal theoretical (open circuit) voltages of glucose/oxygen. (**B**) Power transmission from transmitter to receiver on contact lenses—where *V_g_* is voltage gate, *L_s_* is source coil inductance, *L_d_* is device coil inductance, *R_s_* is transmitter resistance, *R_d_* is receiver resistance, *R_g_* is generator resistance, *R_L_* is load resistance, *C_s_* is source capacitor, *C_d_* is device capacitor, *M* is mutual coupling, *K* is the coupling coefficient and *ω* is the angular frequency of transmission. By using the maximum power transfer theorem, the ratio of load power (*P_L_*) to maximum output power (*P_g,max_*) available to the contact lenses can be given by the equation shown.

Subcellular entities or whole cells are often used as the substrate to convert chemical energy into electrical energy. Microorganisms could play that role by degrading organic materials to produce electrons that will travel to the cathode side via an electronic circuit [45] (Figure 5A). So far, some promising methods have been developed, despite many more problems requiring an efficient solution. In their work, Falk et al. [39] tried to calculate the amount of recoverable energy produced by biofuel cells through human lacrimal fluids. Corynascus thermophilus cellobiose dehydrogenase and myrothecium verrucaria bilirubin oxidase were deployed as efficient and stable anodic and cathodic bioelements, respectively. Analysis of the electrochemical process showed an exchange of electrons from the anode to the cathode resulting from the oxidation of glucose, i.e., production of current.

Reid et al., worked in an open circuit voltage (OCV) of 0.52 V, with a maximum power density of 61 µW/cm^2^ which showed a deviation from the actual results which they described on their graphs. CLs tested in synthetic lacrimal fluids revealed instability of potential and power density, respectively. Practically, the experiment yielded an OCV of 0.41 V, and a current density of 61.3 µA/cm^2^ [37]. The reaction contained the oxidation of lactate at the anode, contrasting with the approach of Falk et al., who utilized glucose [39]. The energy gap between the two approaches is significant. The anode contained nicotinamide adenine dinucleotide using pyrroloquinoline quinone glucose dehydrogenase and a cathode of bilirubin oxidase. After applying a constant voltage of 0.2 V and measuring the current for 17 h, it was estimated that the loss rate reached 80% within the first 4 h.

This serious limitation requires the development of boost converters in the limited area of 0.88 mm^2^ or a series of biofuel cell units [38,46]. Instability is another major challenge for a biofuel cell-powered CL. The electrodes are made of carbon nanotubes that do not provide protection inside the eye. Therefore, it is important, in addition to the above limitations, to focus on developing an extra layer to protect the anterior eye from irritation [37].

Except for biofuel cells, Takamatsu et al. [47] proposed a combination of a Zn-air battery and wireless powering system. The Zn anode acts as antenna and battery at the same time. Different combinations of metals have been carried out to make an optimal voltage enhancement of the cell. This hybrid system of a Zn battery may constitute a viable solution of powering in wireless applications.

### 5.3. Solar Cell

The possibility of powering a CL through solar power has been scarcely reported in the literature. Most of the arguments against this methodology have to do with the indoors generation of power, night-time power generation, and the fact that the CLs do not receive any light when the eyelids are closed [33]. To tackle some of these obvious challenges, Lingley et al. [40] proposed the use of this methodology as a backup for the more popular RF power, additionally noting that the ambient light indoors is sufficient to generate the µW needed by most CL control circuitry. In their work, single diffusion and one metallization was used to fabricate a free-standing 2 × 12 micro-solar cell which was integrated into a CL. Their solar cell had dimensions of 500 × 500 × 10 µm^3^ with a power conversion efficiency of 1.24%, an output voltage of 0.31 V, a short circuit current of 11.5 µA, and a peak responsiveness (absorption) of 725 nm. However, radio and control circuitry and a biosensor had not been incorporated on the CL for a holistic analysis. The authors’ claimed the low-power conversion efficiency was caused by unoptimized doping levels, geometry, and surface passivation. Additionally, the experiment showed an inconsistent power supply which is a problem that can be solved with the use of energy storage components such as supercapacitors [33]. The application of solar power onto CLs is intellectually stimulating, nevertheless many more aspects validating its applicability should first be clarified.

### 5.4. Inductive Power

The general idea behind this methodology lies in the phenomenon of magnetic induction for power transmission. A coupled resonator consists of a primary coil and a secondary coil without direct electrical connection between them. So, it is possible to transmit power using non-irradiated fields at close range. The development of such a device in biomedicine is imminent. From the theoretical perspective, the equivalent circuit of the energy transfer mode is shown in Figure 5B. Aiming at increasing the overall efficiency, the parasitic resistance should be decreased, but this will ordinarily mean an increase in the coupling coefficient and a decrease in the transmitter and receiver distance. This has been one of the many challenges of this approach, because a decrease in transmitter and receiver distance usually reduces flexibility, elegance, and user appreciation. However, in a recent white paper report, a way around this problem was proposed. In the report, it was suggested that by designing a high-quality factor resonator, an efficient energy transfer with a low coupling coefficient could be achieved [48]. Takamatsu et al. recently designed an antenna and a coupling capacitor with a high-power transfer efficiency at a very low frequency. In comparison to other similar approaches, it provides sufficient efficiency relative to the frequency of transmission. Power transfer efficiency barely becomes non-linear as it increases, but the grade is extremely low [49].

Moreover, in terms of soft CLs, the practical foundation of inductive power application dates back to the work of Kenyon at MIT in 1985 [41]. In his work, he sandwiched a coil between two soft CLs and adhered it to the eye using distilled water. The experiment was used to measure eye movement, noting that the signal generated is proportional to the sine of the eye position. The experiment reported corneal deformation and a decrease in intraocular pressure as side-effects. However, in a more recent work, an inductive power approach was used to power CLs to turn on and off an embedded LED, once a glucose threshold was reached [33]. The experiment was carried out in vivo, using rabbits, and yielded a 21.5% power transfer efficiency, an output power of 149.3 mW, and an output voltage and current of 10 V and 14.92 mA, respectively. However, this approach had to deal with the challenge of an uncomfortable 5 mm maximum distance requirement between the transmitter and the receiver coils. Another issue relates to the CL haziness and the possibility of eye irritation; Park et al. [33] mitigated haziness by using highly transparent and stress-tunable hybrid structures with a matched refractive index for clarity. Additionally, they reported the use of 1d ultra-long metal nanofibers and stretchable electrodes as antenna material to overcome mechanical buckling and eye irritation through device stretchability. Earlier, the use of integration-friendly lens coils for overcoming the problem of warpage, wrinkles and irritation had been proposed [42].

With more research towards increasing the distance between the transmitter and receiver, and decreasing the associated discomfort, this methodology has the prospect to eliminate the need for expensive and bulky interrogators by using wireless pixel display indicators.

## 6. Challenges in the Development of Glucose-Sensing CLs

### 6.1. Calibration Issues

As it is vital for every biosensor to provide reliable measurements, the process of calibration in glucose sensing CLs is controversial and still requires many improvements [22]. It varies from device to device as specifications of manufacturers varies and is also affected by the biosensor fabrication materials and processes, and the glucose detection method [23,32,50,51].

### 6.2. Commercial Aspects

The use of CLs as biosensors seems to be a viable strategy in financial terms; nowadays, CLs constitute one of the most popular wearable devices for vision improvement and aesthetic purposes. Moreover, significant progress in the fabrication process and materials used has increased their accessibility and appeal, leading to a dramatic reduction in their cost (daily disposable CLs currently cost 1–2 euros). Accordingly, the systemic use of CLs as biosensors is expected to dramatically reduce their cost as well. At the same time, CLs could potentially record glucose levels for hours, or even days if required. Furthermore, the development of multi-purpose CLs (for instance as dual biosensor–aesthetic devices) could facilitate their marketization potential [52]. Finally, no additional health hazards have been reported for CLs acting as glucose biosensors, aside from those that apply to commercially available CLs (mainly bacterial contamination) [53]. Nevertheless, as this technology is still relatively new and thus mainly experimental, more trials are required to gain a clearer view on potential health hazards, and careful business plan projections are needed to marketize it.

### 6.3. Recent and Future Applications

Recent advances in the development of smart contact lenses for biomedical purposes combine experimentally both continuous glucose monitoring with an on-demand flexible drug delivery system for the treatment of diabetic retinopathy [51]. Future applications could try to introduce multi-purposing CLs which would combine different CL applications, including (aside from glucose sensing capacity) UV protection [54], stem cell transfer into damaged cornea [55,56], restoring visual acuity and/or enhancing human vision (telescopic vision system, augmented/virtual reality system) [57,58,59].

## 7. Conclusions

**Figure 6:**
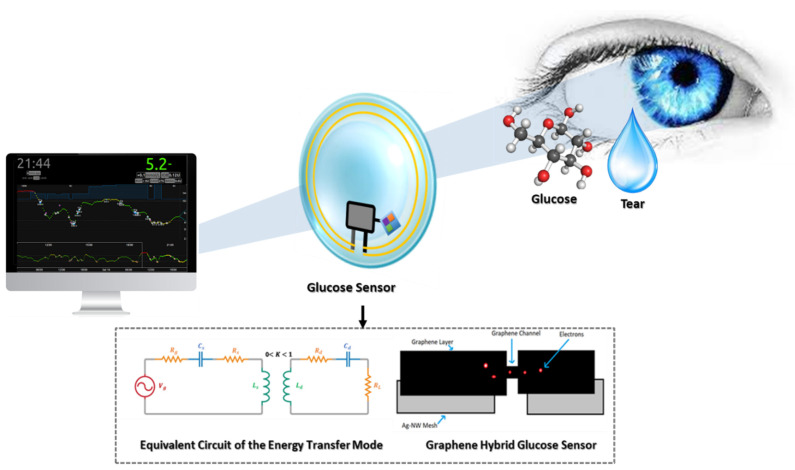
Summary of the key enabling technologies involved in the dynamic, contact lens-mediated glucose sensing process.

Dynamic monitoring of glucose levels allows for tighter control of the hormonal oscillations, improves the therapeutic management of DM and slows the progression of any complications (Figure 6). Various sources of evidence indicate that non-invasive glucose sensors show significant promise on this matter. Graphene-AgNW hybrid sensors, as well as amperometric sensors, have been proven to work as expected through in vivo, preclinical experimentation. The fabrication of an amperometric sensor is comparatively easier and cheaper than a graphene-AgNW hybrid sensor. The fabrication of a graphene-AgNW hybrid entails preparing graphene and silver nanowires separately and then fabricating the actual CL, whereas the amperometric sensor is directly heat-molded with electrodes on the surface of a CL. Once the detection is done through any of these methods, the sensor reading must be processed and transmitted outside the CL. The advancement in IC lithography has given IC manufacturers the ability to mass-produce transistors in sizes as small as 10 nm by 7 nm. Engineers can now pack them in an area as small as 2 × 2 mm^2^, with tiny low microcontrollers coupled with RF data transceivers capable of transmitting data to distances of a few centimeters. Research in materials technology has proven that graphene can provide EMI protection and has a good electrical conductivity, thereby making the composition of bio-compatible devices, such as CL biosensors, possible. Additionally, from the reported review, it is evident that RF is the most popular method of powering CL used for continuous glucose monitoring, as it has been most intensively researched, whereas solar power has the least research coverage so far. It is expected that the development of CL glucose sensors will continue to grow and soon there will be an increase in the availability of commercial products [60].

## References

[1] Guariguata L., Whiting D.R., Hambleton I., Beagley J., Linnenkamp U., Shaw J.E. (2013). Global estimates of diabetes prevalence for 2013 and projections for 2035. Diabetes Res. Clin. Pract..

[2] Roglic G. (2016). WHO Global Report on Diabetes: A Summary. Int. J. Noncommunicable Dis..

[3] Long N.A., Dagogo-Jack S. (2011). The Comorbidities of Diabetes and Hypertension: Mechanisms and Approach to Target Organ Protection. J. Clin. Hypertens..

[4] Badugu R., Lakowicz J.R., Geddes C.D. (2003). Glucose Sensing Contact Lens: A Non-Invasive Technique for Continuous Physiological Glucose Monitoring. J. Fluoresc..

[5] Villiger M., Stoop R., Vetsch T., Hohenauer E., Pini M., Clarys P., Rereira F., Clijsen R. (2017). Evaluation and review of body fluids saliva, sweat and tear compared to biochemical hydration assessment markers within blood and urine. Eur. J. Clin. Nutr..

[6] Choy C.K., Cho P., Chung W.Y., Benzie I.F. (2001). Water-Soluble Antioxidants in Human Tears: Effect of the Collection Method. Investig. Ophthalmol. Vis. Sci..

[7] Zhang J., Hodge W., Hutnick C., Wang X. (2011). Noninvasive Diagnostic Devices for Diabetes through Measuring Tear Glucose. J. Diabetes Sci. Technol..

[8] March F.W. (2001). A Noninvasive Ocular Glucose Sensor. Diabetes Technol. Ther..

[9] Domschk A., March W.F., Kabilan S., Lowe C. (2006). Initial Clinical Testing of a Holographic Non-Invasive Contact Lens Glucose Sensor. Diabetes Technol. Ther..

[10] Kim J., Kim M., Lee M.S., Kim K., Ji S., Kim Y.T., Park J., Na K., Bae K.H., Kim H.K. (2017). Wearable smart sensor systems integrated on soft contact lenses for wireless ocular diagnostics. Nat. Commun..

[11] Luana V.H., Hanb J.H., Kanga H.W., Lee W. (2019). Ultra-sensitive non-enzymatic amperometric glucose sensors based on silver nanowire/graphene hybrid three-dimensional nanostructures. Results Phys..

[12] Chen H.C., Su W.R., Yeh Y.C. (2020). Functional Channel of SWCNTs/Cu2O/ZnO NRs/Graphene Hybrid Electrodes for Highly Sensitive Nonenzymatic Glucose Sensors. ACS Appl. Mater. Interfaces.

[13] Hussaina S., Akbarc K., Vikramana D., Choia D.C., Kimd S.J., An K.S., Jung S., Jung J. (2015). Highly Sensitive Enzymeless Glucose Sensor Based on 3D Graphene-Cu Hybrid Electrodes. New J. Chem..

[14] Yao H., Shum A.J., Cowan M., Lähdesmäki I., Parviz B.A. (2011). A contact lens with embedded sensor for monitoring tear glucose level. Biosens. Bioelectron..

[15] Yao H., Marcheselli C., Afanasiev A., Lähdesmäki I., Parviz B.A. A soft hydrogel contact lens with an encapsulated sensor for tear glucose monitoring. Proceedings of the 2012 IEEE 25th International Conference on Micro Electromechanical Systems (MEMS).

[16] Yao H., Afanasiev A., Lähdesmäki I., Parviz B.A. A dual microscale glucose sensor on a contact lens, tested in conditions mimicking the eye. Proceedings of the 2011 IEEE 24th International Conference on Micro Electromechanical Systems.

[17] Yao H., Liao Y., Lingley A.R., Afanasiev A., Lähdesmäki I., Otis B.P., Parviz B.A. (2012). A contact lens with integrated telecommunication circuit and sensors for wireless and continuous tear glucose monitoring. J. Micromech. Microeng..

[18] Holt-Hindle P., Nigro S., Asmussen M., Chen A. (2008). Amperometric glucose sensor based on platinum–iridium nanomaterials. Electrochem. Commun..

[19] Elsherif M., Hassan M.U., Yetisen A.K., Butt H. (2018). Wearable Contact Lens Biosensors for Continuous Glucose Monitoring Using Smartphones. ACS Nano.

[20] Lin Y.R., Hung C.C., Chiu H.Y., Chang B.H., Li B.R., Cheng S.J., Yang J.W., Lin S.F., Chen G.Y. (2018). Noninvasive Glucose Monitoring with a Contact Lens and Smartphone. Sensors.

[21] Badugu R., Lakowicz J.R., Geddes C.D. A glucose-sensing contact lens: A new approach to noninvasive continuous physiological glucose monitoring. Proceedings of the Biomedical Optics 2004.

[22] Phan C.M., Subbaraman L., Jones L.W. (2016). The Use of Contact Lenses as Biosensors. Optom. Vis. Sci..

[23] Kim S., Jeon H.J., Park S., Lee D.Y., Chung E. (2020). Tear Glucose Measurement by Reflectance Spectrum of a Nanoparticle Embedded Contact Lens. Nature.

[24] Helwig A.M., Arnold M.A., Small G.W. (2000). Evaluation of Kromoscopy: Resolution of glucose and urea. Appl. Opt..

[25] Oliver N.S., Toumazou C., Cass A.E.G., Johnston D.G. (2009). Glucose sensors: A review of current and emerging technology. Diabet. Med..

[26] Cameron B.D., Anumula H. (2006). Development of a real-time corneal birefringence compensated glucose sensing polarimeter. Diabetes Technol. Ther..

[27] Park J., Kim J., Kim S.Y., Cheong W.H., Jang J., Park Y.G., Na K., Kim Y.T., Heo J.H., Lee C.Y. (2018). Soft, smart contact lenses with integrations of wireless circuits, glucose sensors, and displays. Sci. Adv..

[28] Leonardi M., Pitchon E.M., Bertsch A., Renaud P., Mermoud A. (2009). Acta Ophthalmologica.

[29] Craighead H.G., Cheng J., Hackwood S. (1982). New display based on electrically induced index-matching in an inhomogeneous medium. Appl. Phys. Lett..

[30] Lee S., Jo I., Kang S., Jang B., Moon J., Park J., Lee S., Rho S., Kim Y.I., Hong H.B. (2017). Smart contact lenses with Graphene Coating for Electromagnetic Interference Shielding and Dehydration Protection. ACS Nano.

[31] Pandey J., Liao Y.T., Lingley A., Mirjalili R., Parviz B., Otis B.P. (2010). A Fully Integrated RF-Powered Contact Lens with a Single Element Display. IEEE Trans. Biomed. Circuits Syst..

[32] Liao Y.T., Yao H., Lingley A., Parviz B., Otis B.P. (2012). A 3-μW CMOS Glucose Sensor for Wireless Contact-Lens Tear Glucose Monitoring. IEEE J. Solid-State Circuits.

[33] Blum Z., Pankratov D., Shleev S. (2014). Powering electronic contact lenses: Current achievements, challenges, and perspectives. Expert Rev. Ophthalmol..

[34] IEEE International Committee on Electromagnetic Safety (SCC39) (2006). IEEE Standard for Safety Levels with Respect to Human Exposure to Radio Frequency Electromagnetic Fields, 3 kHz to 300 GHz.

[35] Cheng H.W., Jeng B.M., Chen C.Y., Huang H.Y., Chiou J.C., Luo C.H. The rectenna design on contact lens for wireless powering of the active intraocular pressure monitoring system. Proceedings of the 35th Annual International Conference of the IEEE Engineering in Medicine and Biology Society (EMBC).

[36] Chiou J.C., Hsu S.H., Liao Y.T., Huang Y.C., Yeh G.T., Kuei C.K., Dai K.S. (2016). Toward a Wirelessly Powered On-Lens Intraocular Pressure Monitoring System. IEEE J. Biomed. Health Inform..

[37] Reid R.C., Minteer S.D., Gale B.K. (2015). Contact lens biofuel cell tested in a synthetic tear solution. Biosens. Bioelectron..

[38] Kulkami T., Slaughter G. Simulatenous glucose sensing and powering of glucometer. Proceedings of the IEEE 60th International Midwest Symposium on Circuits and Systems (MWSCAS).

[39] Falk M., Andoralov V., Blum Z., Sotres J., Suyatin D.B., Ruzgas T., Arnebrant T., Shleev S. (2012). Biofuel cell as a power source for electronic contact lenses. Biosens. Bioelectron..

[40] Lingley A.R., Otis B.P., Shen T.T., Parviz B.A. (2012). A contact lens with integrated micro solar cells. Microsyst. Technol..

[41] Kenyon R.V. (1985). A soft contact lens search coil for measuring eye movements. Vis. Res..

[42] Chen L., Shaker G., Safavi-Naeini S. Warpage-free antenna for smart contact lens applications. Proceedings of the 2017 IEEE International Symposium on Antennas and Propagation & USNC/URSI National Radio Science Meeting.

[43] Friis H.T. (1946). A Note on a Simple Transmission Formula. Proc. IRE.

[44] Chen L., Shaker G., Safavi-Naeini S. Energy harvesting system integrated on wearable contact lens. Proceedings of the 2015 IEEE International Symposium on Antennas and Propagation & USNC/URSI National Radio Science Meeting.

[45] He Z., Angenent L.T. (2006). Application of Bacterial Biocathodes in Microbial Fuel Cells. Electroanalysis.

[46] Carreon-Bautista S., Erbay C., Han A., Sanchez-Sinencio E. (2015). An Inductorless DC–DC Converter for an Energy Aware Power Management Unit Aimed at Microbial Fuel Cell Arrays. IEEE Trans. Emerg. Sel. Top. Power Electron..

[47] Takamatsu T., Sijie Y., Shujie F., Xiaohan L., Miyake T. (2019). Multifunctional High-Power Sources for Smart Contact Lenses. Adv. Funct. Mater..

[48] Takamatsu T., Chen Y., Yoshimasu T., Nishizawa M., Miyake T. (2019). Highly Efficient, Flexible Wireless-Powered Circuit Printed on a Moist, Soft Contact Lens. Adv. Mater. Technol..

[49] Kesler M. (2017). Highly Resonant Wireless Power Transfer: Safe, Efficient, and Over Distance.

[50] Lodwig V., Heinemann L. (2003). Continuous Glucose Monitoring with Glucose Sensors: Calibration and Assessment Criteria. Diabetes Technol. Ther..

[51] Keum D.H., Kim S.K., Koo J., Lee G.H., Jeon C., Mok J.W., Mun B.H., Lee K.J., Kamrani E., Joo C.K. (2020). Wireless smart contact lens for diabetic diagnosis and therapy. Sci. Adv..

[52] Tseng R.C., Chen C.C., Hsu S.M., Chuang H.S. (2018). Contact-Lens Biosensors. Sensors.

[53] Moreddu R., Vigolo D., Yetisen A.K. (2019). Contact Lens Technology: From Fundamentals to Applications. Adv. Healthc. Mater..

[54] Behar-Cohen F., Baillet G., de Ayguavives T., Garcia P.O., Krutmann J., Peña-García P., Remé C., Wolffsohn J.S. (2014). Ultraviolet damage to the eye revisited: Eye-sun protection factor (E-SPF^®^), a new ultraviolet protection label for eyewear. Clin. Ophthalmol..

[55] Di Girolamo N., Bosch M., Zamora K., Coroneo M.T., Wakefield D., Watson S.L. (2009). A contact lens-based technique for expansion and transplantation of autologous epithelial progenitors for ocular surface reconstruction. Transplantation.

[56] Bobba S., Di Girolamo N. (2016). Contact Lenses: A Delivery Device for Stem Cells to Treat Corneal Blindness. Optom. Vis. Sci..

[57] Lan S., Zhang X., Taghinejad M., Rodrigues S., Lee K.T., Liu Z., Cai W. (2019). Metasurfaces for Near-Eye Augmented Reality. ACS Photon..

[58] Augmented-Reality Contact Lenses to be Human-Ready at CES. http://www.cnet.com/news/augmented-reality-contact-lenses-to-be-human-ready-at-ces/.

[59] Glenn M.S., Arianpour A., Cookson S., Zhang A., Hendrik L., O’Brien T., Alvarez A., Ford J.E. (2015). Wink-controlled polarization-switched telescopic contact lenses. Appl. Opt..

[60] Ascaso F.J., Huerva V. (2016). Noninvasive Continuous Monitoring of Tear Glucose Using Glucose-Sensing Contact Lenses. Optom. Vis. Sci..

